# Effects of dietary supplementation of creatine on fetal development in gilts at d 60 and d 90 of gestation

**DOI:** 10.1186/s40104-025-01166-0

**Published:** 2025-03-01

**Authors:** Arianna N. Lopez, Maddison A. Olivarez, Claire Stenhouse, Robyn M. Moses, Makenzie G. Newton, Nirvay Sah, Heewon Seo, Joseph Cain, Carli Lefevre, Alexandria Ross, Patrick Ryan, Jeffrey G. Wiegert, Guoyao Wu, Gregory A. Johnson, Fuller W. Bazer

**Affiliations:** 1 Department of Animal Science, Texas A&M University, College Station, TX 77843 USA; 2 Department of Animal Science, Pennsylvania State University, University Park, PA 16802 USA; 3 Department of Obstetrics and Gynecology, University of Nebraska Medical Center, Omaha, NE 68198 USA; 4 Department of Pathology, University of California-San Diego, San Diego, CA 92093 USA; 5 Department of Veterinary Integrative Biosciences, Texas A&M University, College Station, TX 77843 USA; 6 Department of Animal and Avian Sciences, University of Maryland, College Park, MD 20742 USA; 7 Department of Kinesiology, Texas A&M University, College Station, TX 77843 USA

**Keywords:** Creatine, Development, Fetal, Gestation, Supplementation

## Abstract

**Background:**

The creatine-creatine kinase-phosphocreatine (Cr-CK-PCr) system maintains intracellular ratios of ATP/ADP for support of cellular functions and has been characterized at the placental-uterine interface of rodents, primates, swine and sheep, and thus may support fetal development. This study determined effects of dietary supplementation of creatine (Cr) to gestating gilts on fetal development, the number and ratio of primary and secondary muscle fibers, and on protein expression in endometrium and fetal biceps-femoris muscle, respectively in fetal pigs on d 60 and d 90 of gestation.

**Methods:**

Reproductively mature gilts were synchronized to estrus using Matrix, observed for estrus (d 0), and artificially inseminated 12 h and 36 h later. Gilts were individually housed and fed 0.86 kg of 14% crude protein diet twice daily that meets nutritional requirements for pregnant gilts. Gilts were assigned to either basal diet control (CON) group, or Cr supplemented group (provided 30 g Cr monohydrate daily) from d 10 to either d 60 or d 90 of gestation. Gilts were euthanized and hysterectomized on either d 60 or d 90 of gestation. These protocols were completed in two replicates, as gilts were bred in spring and euthanized in summer or bred in fall and euthanized in winter (*n* = 20 gilts/replicate). Litter size, crown-rump length, sex, and fetal weight was recorded. Three female and male fetuses closest to mean litter weight were selected to assess effects of treatment on weight of fetal brain, kidney, liver, spleen, and biceps-femoris muscle. Data were analyzed to determine effects of treatment, days of gestation, replicate, and sex on litter size, fetal measurements, and incidence of intrauterine growth restriction.

**Results:**

Dietary Cr supplementation increased fetal brain weight to body weight ratios on d 90 of gestation (*P* < 0.05) and fetal kidney weight to body weight ratios on d 60 of gestation (*P* < 0.01), while days of gestation had significant effect on expression of mitochondrial CK isoform in gilt endometria (*P* $<$ 0.05).

**Conclusions:**

Results suggest that dietary supplementation of Cr in gestating gilts enhanced development of select fetal organs and contribute to understanding roles of the Cr-CK-PCr system in pregnancy.

**Supplementary Information:**

The online version contains supplementary material available at 10.1186/s40104-025-01166-0.

## Introduction

The term intrauterine growth restriction (IUGR) refers to impaired growth and development of a mammalian embryo, fetus, and/or fetal organs during pregnancy [1]. IUGR occurs in 15%–20% of newborn piglets and the incidence of IUGR reduces neonatal survival, postnatal growth, nutrition utilization, and health of piglets [1]. IUGR piglets at birth and at 150 days of age also have a lower ratio of muscle fibers to connective tissue in their bodies [2]. Collectively, these negative impacts of IUGR cause severe economic losses in the swine industry, therefore it is important to prevent and correct effects in growing animals [1]. Diagnosis of IUGR is based on a determination that a neonate’s birth weight is lower than the two standard deviations of mean birth weight for breed and gestational age [3]. Both biological and environmental factors contribute to IUGR, including but not limited to genetics and maternal maturity [3]. However, maternal nutrition, placental efficiency, and uterine capacity have also been demonstrated to affect fetal growth and development [3]. As IUGR has severe implications for animal agriculture, the underlying mechanisms for nutritional effects on growth and developmental restriction should be investigated to provide insight for development of strategies to prevent and mitigate its occurrence. Alterations to maternal nutrition has been heavily studied and may be effective in increasing uniformity among conceptuses (embryo/fetus and extra-embryonic/placental membranes) [3, 4], because both fetal and maternal metabolic states are vital for a successful pregnancy and the overall success of the swine industry.

As a lower ratio of muscle fibers to connective tissue within fetal bodies has been associated with the incidence of IUGR, appropriate fetal muscle fiber development is vital for fetal growth, and postnatal outcomes [2]. Myofibers are formed from myoblast (myogenic precursor cell) fusion into multinucleated myotubes that develop into myofibers and later into muscle fibers [5, 6]. In swine fetuses, primary muscle fiber formation is complete by d 60 of gestation, and secondary fiber formation is complete by d 90 of gestation [7]. Once primary muscle fibers have formed, secondary muscle fibers use the primary fibers as a scaffold around which to develop. The contractile velocity varies from muscle to muscle and is classified as either fast-twitch (glycolytic) or slow-twitch (oxidative) fibers [8]. The myosin heavy chains (MyHC) that form myofibers are classified as either “fast” twitch MyHCIIa, MyHCIIb, and MyHCIIx, or “slow” twitch MyHCI [8].

**Fig. 1:**
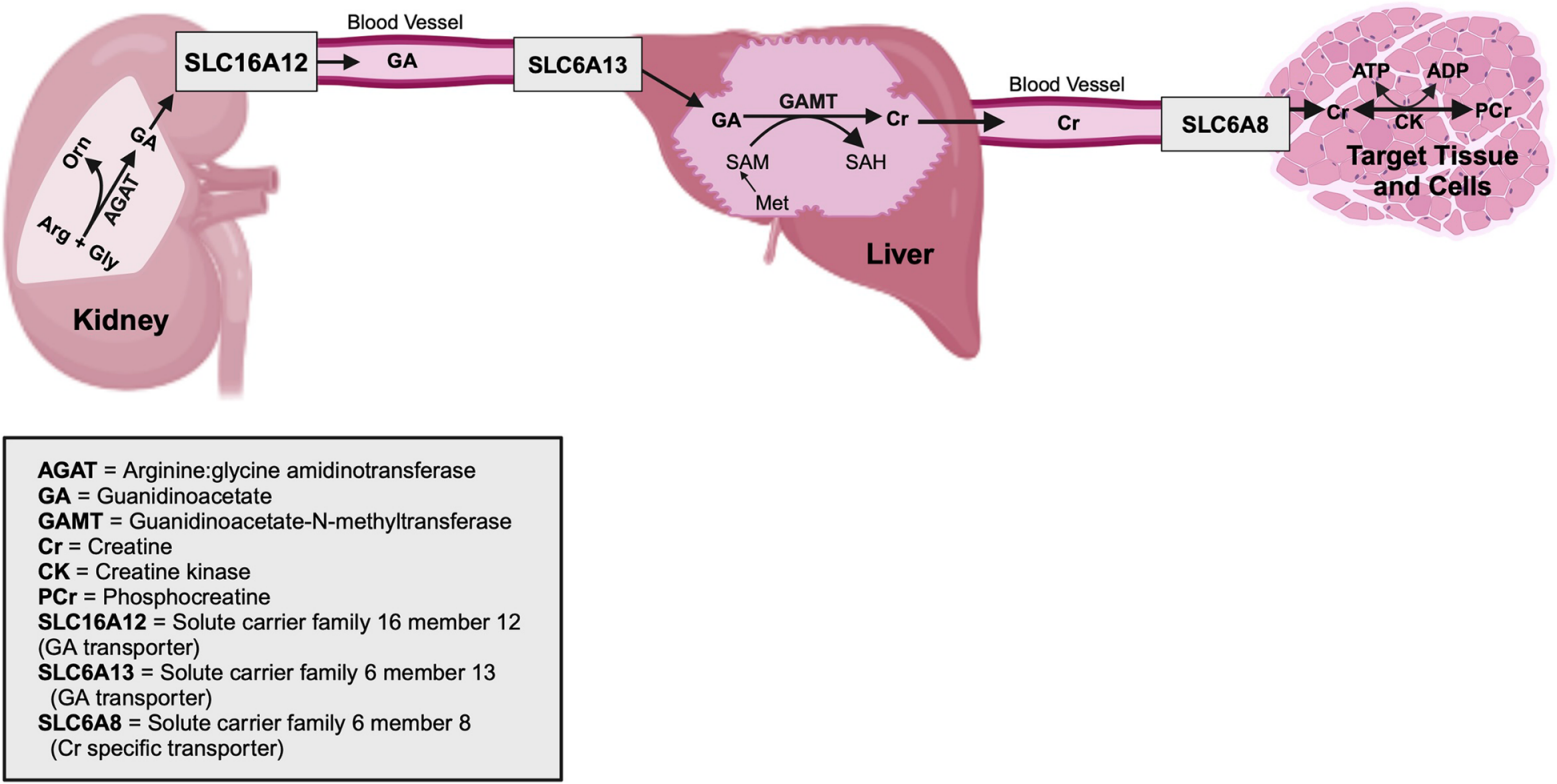
Pathway for synthesis of creatine (Cr). Synthesis of guanidinoacetate (GA) by arginine:glycine amidinotransferase (AGAT) from arginine (Arg) and glycine (Gly) occurs in the kidney and GA is transported to the cells of the liver via solute carriers Cr transporter 2 (SLC16A12) and GAT2 (SLC6A13). In the cells of the liver, GA is converted to creatine (Cr) by guanidinoacetate-*N* methyltransferase (GAMT) and Cr is transported to target tissues/cells by SLC6A8. The liver of pigs has a low activity of Cr kinase and thus releases most Cr to the blood. In extrahepatic tissues and cells, Cr is converted to creatine phosphate [also known as phosphocreatine (PCr)] by isoforms of creatine kinase (CK) for the regeneration of adenosine triphosphate (ATP) from adenosine diphosphate (ADP). Created with BioRender.com

The creatine-creatine kinase-phosphocreatine (Cr-CK-PCr) system has vital roles in skeletal muscle as it maintains intracellular ratios of ATP/ADP for support of multiple cellular functions in tissues with high and fluctuating demands for ATP [9]. The Cr-CK-PCr system rapidly regenerates ATP as creatine kinase (CK) and its isoforms such as brain-type creatine kinase (CKB), muscle-type creatine kinase (CKM), and mitochondrial creatine kinases 1 and 2 (CKMT1/CKMT2) reversibly convert Cr and creatine phosphate (PCr), which then donates a phosphate group to adenosine diphosphate (ADP) to regenerate ATP [9]. Muscle contractions cannot occur when CK is inhibited, thus emphasizing the importance of the Cr-CK-PCr system in supporting muscle function [10]. Postnatally, Cr synthesis begins primarily in the kidneys, with arginine and glycine being converted into guanidinoacetate (GA) via arginine-glycine amidinotransferase (AGAT) [11]. GA is then released from the kidneys into the vasculature and transported into cells of the liver and possibly other organs for methylation by guanidinoacetate*-N-*methyltransferase (GAMT) into Cr. Subsequently, Cr is released into the vasculature, and a specific Cr transporter, solute carrier family 6 member 8 (SLC6A8), mediates transport of Cr into target cells [12]. The postnatal Cr synthesis pathway is outlined in Fig. 1. As the Cr-CK-PCr system’s ATP buffering capacity is important for muscle cell function, it may also be involved in fetal muscle development. A previous study reported that the formation of secondary muscle fibers may be highly susceptible to external influences, such as nutrition, in fetal piglets [13]. As the fetus depends upon the maternal plane of nutrition for proper growth, it also depends on maternal supplies of Cr while developing [14]. It has been reported that Cr may aid in maintaining ionic balance and promote muscle protein synthesis, it may hold important roles in fetal muscle fiber development [14]. This study tested the hypothesis that dietary supplementation of Cr to gestating gilts may improve fetal development via enhancing the Cr-CK-PCr system’s roles in fetal and maternal tissues. Thus, we determined the effects of dietary supplementation of Cr to gestating gilts on fetal growth and development including primary and secondary skeletal muscle fiber development in fetal pigs at d 60 and d 90 of gestation.

## Materials and methods

### Experimental animals and sample collection

All experimental procedures followed the Guide for the Care and Use of Agriculture Animals in Research and Teaching and were approved by the Institutional Animal Care and Use Committee of Texas A&M University. Sexually mature gilts that had experienced at least two estrous cycles of normal duration were synchronized to estrus using Matrix (Altrenogest; Merck, Rahway, NJ, USA), a synthetic progestin. Matrix was administered (6 mL) top dressed on feed once daily for 14 d. Gilts were observed for onset of estrus behavior (standing heat) twice daily following removal of Matrix supplementation. Once onset of estrus was detected (designated d 0), the gilts were artificially inseminated 12 h and 36 h later. All gilts were individually housed from d 9 of gestation and fed 0.86 kg corn- and soybean-based diet containing 14% crude protein twice daily (1.72 kg diet/d). The composition of the basal diet is given in Table 1. Gilts were assigned randomly to either control (CON) or creatine (Cr) treatment groups and to be euthanized on either d 60 or d 90 of gestation. A daily dietary supplement of 30 g Cr monohydrate (Pure Bulk, Roseburg, OR; 15 g twice daily) was provided to pregnant gilts assigned to the Cr treatment group from d 10 of gestation to either d 60 or d 90 of gestation. The dosage of 30 g Cr monohydrate daily was selected based on a dose-dependent study revealing that doses ranging from 25 to 50 g Cr monohydrate administered daily to Duroc and Landrace pigs increased the concentrations of Cr in plasma [15]. Gilts were fed their assigned treatment diets until either d 60 or d 90 of gestation as the maximum number of primary muscle fibers is established by d 60, and the maximum number of secondary muscle fibers is established by d 90 of gestation. On either d 60 or 90 of gestation, a blood sample was collected from gilts prior to euthanasia and hysterectomy. A blood sample was collected from gilts into 6 mL BD vacutainer tubes (Fisher Scientific, Waltham, MA, USA), then were centrifuged (Eppendorf centrifuge 5920R Hamburg, Germany) at 5 °C for 18 min at 2,600 × *g* and plasma was harvested and stored at −20 °C.

**Table 1:** Nutrient levels of the basal diet^a^

Nutrient	Content in diet
Crude protein, %	14.0
Metabolizable energy, MJ/kg	14.3
Lysine, %	0.63
Crude fat, %	3.0
Crude fiber, %	4.0
Calcium, %	0.90
Total phosphorous, %	0.78
Sodium, %	0.18
Chlorine, %	0.68
Potassium, %	1.0
Vitamin premix, %^b^	0.30
Mineral premix, %^c^	0.10

^a^The dietary ingredients (%, as-fed basis) are as follows: corn grain, 79.85; soybean meal (48% crude protein), 15.0; monocalcium phosphate, 2.0; potassium chloride, 0.8; ground limestone, 1.0; soybean oil, 0.55; salt mix, 0.4

^b^The vitamin premix provided the following (mg/kg of the basal diet): retinyl acetate, 9.0; cholecalciferol, 0.055; D-α-tocopheryl acetate, 71; menadione sodium bisulfate, 2.0; choline, 1,230; riboflavin, 9.5; niacin, 73; pantothenic acid, 38; vitamin B_12_, 0.045; biotin, 0.26; vitamin B_6_, 8.8; and thiamine, 5.0

^c^The mineral premix provided the following (mg/kg of the basal diet): manganese: 50; iron, 253; copper, 25; cobalt, 0.17; iodine, 0.68; zinc, 198; selenium, 0.30

The experimental protocols were completed in two replicates (*n* = 20 gilts/replicate). The first replicate was completed beginning with gilts synchronized to estrus with Matrix starting on April 4, 2023, and inseminated artificially beginning on April 23, 2023 following onset of estrus (designated d 0). We had two replicates of 20 gilts each because we did not have facilities to manage more than 20 gilts per replicate. One replicate was conducted in the Fall and one replicate was conducted in the Summer. The randomly assigned treatment diets were started on d 10 of gestation for each gilt. The first replicate of gilts began with treatment diets on May 2, 2023 and euthanized in either June or July 2023, respectively on either d 60 or 90 of gestation. The second replicate began with gilts synchronized to estrus starting on September 21, 2023, and inseminated beginning on October 9, 2023 following onset of estrus (designated d 0). The seasons in which these replicates were completed were selected with aims to avoid heat stress on the gestating gilts. The randomly assigned treatment diets were started on d 10 of gestation for each gilt. The second replicate began on October 19, 2023, and gilts were euthanized in either December 2023 or January 2024, respectively on either d 60 or 90 of gestation. Therefore, there were four treatment groups: CON d 60, Cr d 60, CON d 90, and Cr d 90 (*n* = 5 gilts/treatment/day of gestation/replicate).

Data including number of corpora lutea (CL), number of live, dead, and mummified fetuses, and total litter size were recorded. Samples of endometrium were dissected and frozen in liquid nitrogen for analyses using Western blotting to determine the expression of candidate proteins of interest. Fetal crown-rump length (CRL) and body weight were recorded to assess effects of treatment on fetal development and litter weight. Based on mean body weight within each litter, three male and three female fetuses closest to the mean litter weight were selected and blood samples collected in 6-mL BD vacutainer blood collection tubes (Fisher Scientific, Waltham, MA, USA) from the umbilical cord. Blood samples were centrifuged (Eppendorf centrifuge 5920R Hamburg, Germany) at 5 °C for 18 min at 2,600 × *g* and plasma harvested and stored at −20 °C. From the selected male and female fetuses, organ weight of brain, kidney, liver, spleen and biceps-femoris muscle of the right hind-limb was recorded to assess effects of treatment on fetal development. Also, from the selected male and female fetuses, the biceps-femoris muscle dissected from the right hind-limb was fixed in 4% paraformaldehyde and paraffin-embedded, while the biceps-femoris muscle from the left hind-limb was frozen in optical cutting temperature (OCT) for muscle fiber type analyses using immunofluorescence analyses, as well as snap frozen in liquid nitrogen for analyses using Western blotting.

### Fetal organ weight, body weight, crown-rump length (CRL) comparisons

For each organ, the ratio of organ weight to body weight was compared across treatments. Treatment effects on fetal body weight and CRL were determined. Each fetus was evaluated for IUGR and classified as IUGR if body weight was at least 2 standard deviations below mean fetal weight for breed and gestational age [3]. The incidence of IUGR was assessed to determine effects of dietary treatment and litter size.

### Western blot analyses

Western blot analyses were performed on protein extracts from frozen endometria and fetal biceps-femoris muscle samples from gilts in the first replicate of this study to detect candidate proteins of interest. Biceps-femoris muscle (300–500 mg) was homogenized in a buffer consisting of 60 mmol/L Tris, 0.5 mol/L Na_3_VO_4_, 10% glycerol, 10% SDS, and protease inhibitor cocktail (Roche, Basel, Switzerland). The protein lysate was transferred to a 1.5-mL tube and centrifuged for 15 min at 14,000 × *g* at 4 °C. The supernatant was transferred to a 1.5-mL tube and protein content quantified using the Bradford protein assay as per the manufacturer’s instructions (Bio-Rad, Hercules, CA, USA). Western blot analyses were completed as previously described [16, 17]. Equal amounts of protein (20 μg) were loaded onto a 4%–20% Mini-PROTEAN TGX Precast Protein Stain-Free Gel (Bio-Rad), and separated at 120 V for 1 d. Following electrophoresis, proteins were exposed to Trans UV light for 1 min. Proteins were then transferred to a nitrocellulose membrane (Trans-Blot Turbo, Bio-Rad) for 30 min at 25 V followed by blocking in 5% fat-free milk prepared in 20 mmol/L Tris, 150 mmol/L NaCl, pH 7.5, and 0.1% Tween-20 (TBST) for 1 d. The membrane was incubated overnight at 4 °C with a primary antibody diluted in 2% fat-free milk in TBST. Primary antibodies utilized were: rabbit polyclonal antibody CKMT1A (15346-1-AP, Protein Tech, Rosemont, IL, USA) at a 1:500 dilution; CKMT2 (ab121450, Abcam, Cambridge, UK) at a 1:500 dilution; CKMM (ab151465, Abcam) at a 1:500 dilution; CKB (ab92452, Abcam) at a 1:500 dilution; and GAMT (ab126736, Abcam) at 1:500 dilution. Subsequently, the membrane was washed in TBST (3 times for 10 min each), then was incubated in secondary antibody at 1:5,000 dilution in 2% fat-free milk in TBST; horseradish peroxidase conjugated anti-rabbit (Cell Signaling, Danvers, MA, USA) for 1 d followed by washing in TBST (3 times for 10 min each) and the membrane was imaged under UV light (302 nm) using a ChemiDoc XRS + Quantity One software (Bio-Rad) to obtain the Stain-Free total protein image as previously described [18]. The membrane was then incubated with SuperSignal West Dura Extended Duration Substrate (ThermoFisher Scientific, Waltham, MA, USA) for 5 min and then imaged using a ChemiDoc EQ system and Quantity One software (Bio-Rad). The intensity of protein bands was quantified using Image J software (v1.53, National Institutes of Health) [19].

### Muscle fiber type analyses

To quantify the primary and secondary fetal skeletal muscle fibers, immunofluorescence was performed on fetal biceps-femoris muscle samples from the first replicate of this study [20]. Briefly, biceps-femoris sections were cut at 8 μm thickness at −20 °C using a Leica CM1520 cryostat (Leica Biosystems, Wetzlar, Germany) and adhered to Superfrost Plus microscope slides (Fisher Scientific, Hampton, NH, USA). Muscle sections were allowed to air dry for 1 h, fixed in acetone at −20 °C for 10 min, and then washed in 1 × PBS (phosphate buffered saline). Blocking of non-specific binding sites was accomplished by incubation in 10% normal goat serum (ThermoFisher Scientific, Waltham, MA, USA) for 15 min at room temperature, and then washed in 1 × PBS. Biceps-femoris sections were incubated overnight at 4 °C with primary antibodies at a 1:500 dilution for myosin heavy chain Type 1 (BA-D5-s, DSHB, Iowa City, IA, USA) to determine primary muscle fibers, and myosin heavy chain Type IIa (SC-71-s, DSHB) to determine secondary muscle fibers. Following washing for 15 min in 1 × PBS, samples were incubated at room temperature (25 °C) for 1 d with secondary antibodies at a 1:200 dilution of goat-anti mouse IgG2b secondary antibody Alexa Fluor 633 (A-21146, ThermoFisher, Waltham MA, USA) and goat anti-mouse IgG1 Cross-Absorbed Secondary Antibody Alexa Fluor 488 (A-21121, ThermoFisher). After washing 15 min in 1 × PBS, slides were air-dried for 15 min, and cover slips affixed with Invitrogen Prolong Gold anti-fade reagent (P10144, ThermoFisher). Images were captured using a Nikon Eclipse microscope and NIS-Elements AR 4.30.02 64-bit Software (Nikon Instruments Inc., Melville, NY, USA) with a 20×/0.45 NA objective lens. For each fetal biceps-femoris muscle sample, five non-overlapping images were taken for counting primary and secondary muscle fibers using ImageJ 1.54 g Java software (National Institutes of Health, Bethesda, MD, USA). The counting of muscle fibers was conducted by one individual who was blind to treatment groups. The mean values of primary and secondary muscle fibers from 5 non-overlapping images for each fetus was then compared to determine effects of treatment.

### Statistical analyses

All statistical analyses were performed using the JMP Pro software version 15.0 (SAS Institute Inc., Cary, NC, USA), and statistical significance was declared at* P* $\le$ 0.05. The distribution of data was assessed for normality via a goodness-of-fit test. Data that did not follow a Gaussian distribution were log transformed and checked for normality again. If data were normally distributed, one-way ANOVA for mean comparisons were made. If data were not normally distributed, a nonparametric Wilcoxon/Kruskal–Wallis test was used for mean comparisons. The effects of treatment and litter size on occurrence of IUGR was assessed through a fit-model least squares regression test. Statistical analyses were performed to determine the effects of treatment on fetal organ development, fetal body weight and CRL, and IUGR occurrence. As this study included two replicates (*n* = 20 gilts/replicate; *n* = 5 gilts/treatment/day of gestation/replicate), statistical analyses were completed to determine effects of day and season on fetal development and litter weight.

## Results

**Fig. 2:**
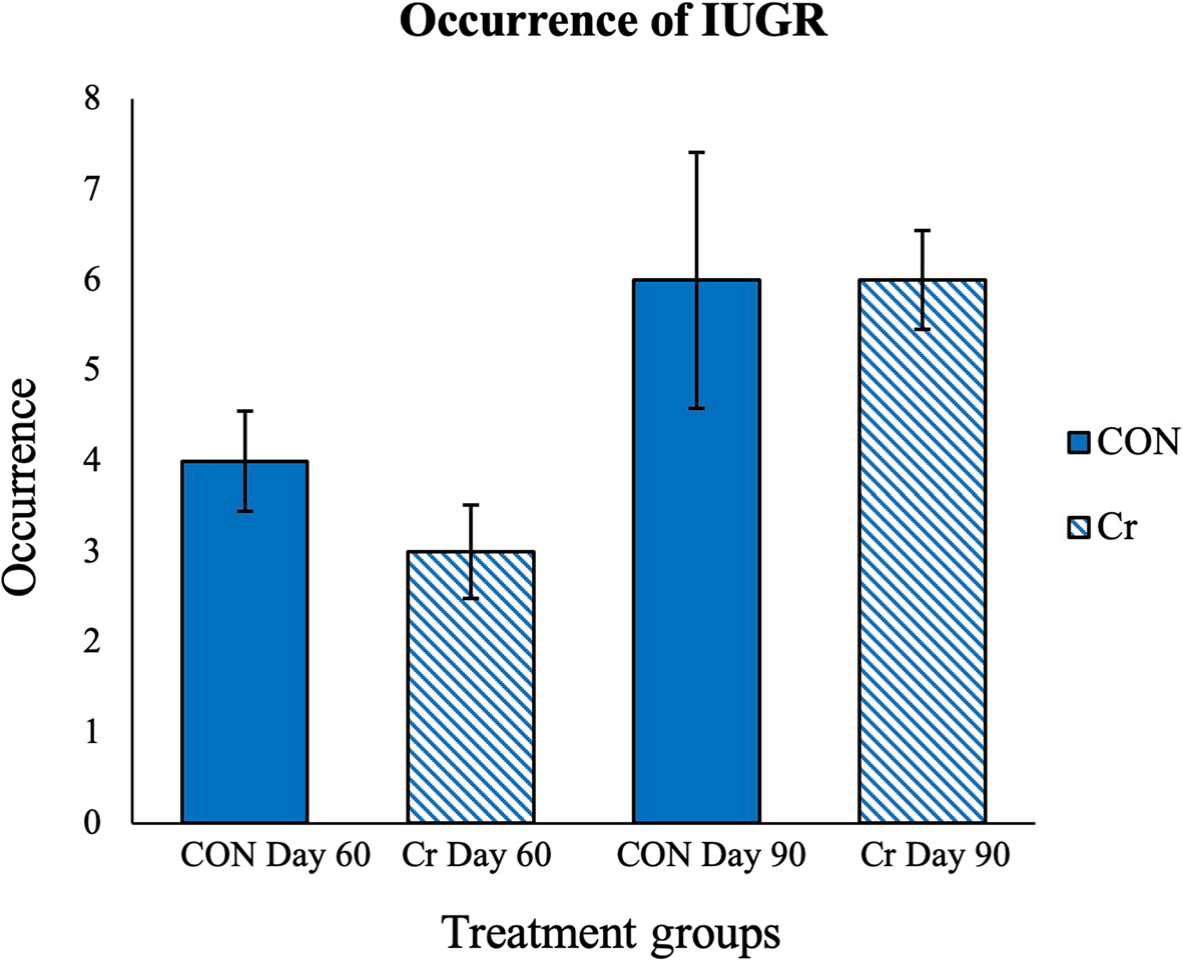
Summary of occurrences of intrauterine growth restriction (IUGR) of fetuses across treatment groups and days of gestation. Values are mean ± SD (*n* = 9 gilts CON d 60, *n* = 10 gilts Cr d 60, *n* = 9 gilts CON d 90, and *n* = 10 gilts Cr d 90; *N* = 225 fetuses)*.* There were 4 occurrences of IUGR in the CON d 60 treatment group, 3 occurrences in the Cr d 90 treatment group, 6 occurrences in the CON d 90 treatment group, and 6 occurrences within the Cr d 90 treatment group. Statistical analyses of occurrences of IUGR detected no effect of treatment (*P* > 0.05)

This study included four treatment groups of (*n* = 9 gilts CON d 60, *n* = 10 gilts Cr d 60, *n* = 9 gilts CON d 90, and *n* = 10 gilts Cr d 90 of gestation) that yielded 225 fetuses total (*n* = 52 CON d 60 fetuses, *n* = 60 Cr d 60 fetuses, *n* = 54 CON d 90 fetuses, and *n* = 59 Cr d 90 fetuses). The pregnancy rates for gilts in the summer and fall replicates of 86% and 90%, respectively, were not different. There was no significant treatment effect on litter size or litter weight (*P* > 0.05), and results for treatment groups, litter size, and litter weight are summarized in Table 2. The total incidence of IUGR within each treatment group was calculated and results are summarized in Fig. 2. There were 4 occurrences of IUGR in the CON d 60 treatment group, 3 occurrences in the Cr d 60 treatment group, 6 occurrences in the CON d 90 treatment group, and 6 occurrences within the Cr d 90 treatment group. Statistical analyses of occurrences of IUGR detected no effects of treatment or litter size (*P* > 0.05).

**Table 2:** Summary of data for treatment groups including both replicates

**Item**	**Treatment** g**roups**			
	**CON Day 60**	**Cr Day 60**	**CON Day 90**	**Cr Day 90**
Sample size, n	9	10	9	10
Mean litter size, n	11$\pm 3.2$	13$\pm 2.7$	14$\pm$1.7	13$\pm 2.3$
Mean number of corpora lutea, n	14$\pm 2.9$	14$\pm 2.3$	16$\pm 2.4$	15$\pm 2.3$
Mean litter weight, g	134.2$\pm 11.9$	129.5$\pm 16.8$	618.2$\pm 71.2$	629.1$\pm 84.2$

### Effects of Cr supplementation on fetal development

**Fig. 3:**
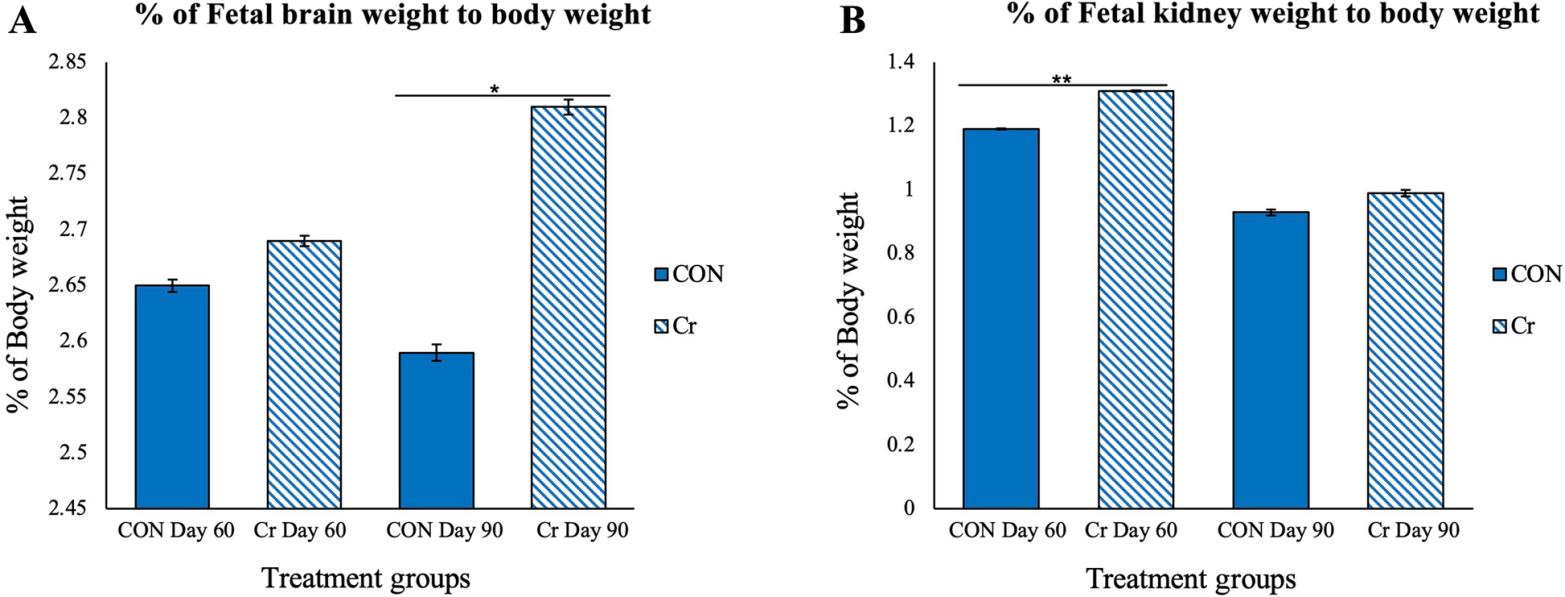
Brain weight to body weight ratios (**A**) and kidney weight to body weight ratios (**B**) among treatment groups. Values are mean $\pm$ SEM (*N* = 225 fetuses; *n* = 52 CON d 60 fetuses, *n* = 60 Cr d 60 fetuses, *n* = 54 CON d 90 fetuses, and *n* = 59 Cr d 90 fetuses). Fetuses from creatine supplemented gilts had heavier brain weight to body weight ratios than those from CON treated gilts on d 90 of gestation (*P* < 0.05). Fetuses from creatine supplemented gilts had greater kidney weight to body weight ratios as compared to those for fetuses from CON gilts on d 60 of gestation (*P* < 0.01)

**Fig. 4:**
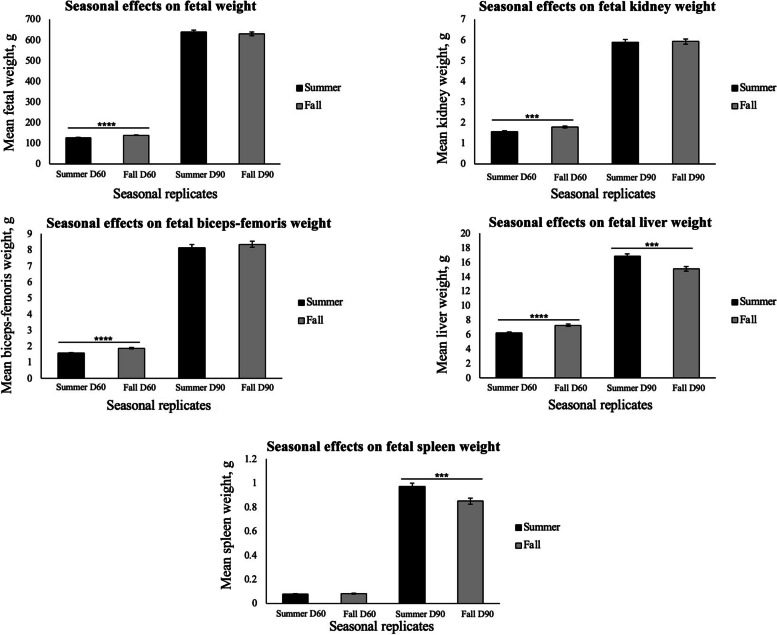
Seasonal effects on mean weight of fetal organs. Values are mean $\pm$ SEM (*N* = 225 fetuses; *n* = 52 CON d 60 fetuses, *n* = 60 Cr d 60 fetuses, *n* = 54 CON d 90 fetuses, and *n* = 59 Cr d 90 fetuses). Mean weight of fetuses, as well as weight of kidney, liver, and biceps-femoris were greater for the fall replicate (replicate 2) than the summer replicate (replicate 1) on d 60 of gestation (*P* < 0.0001). Mean spleen and liver weight for fetuses was greater (*P* < 0.001) in the summer (replicate 1) than the fall (replicate 2) on d 90 of gestation

Fetuses from Cr supplemented gilts had a greater brain weight to body weight ratio than fetuses from CON gilts on d 90 of gestation (*P* < 0.05; Fig. 3A). Fetuses from Cr supplemented gilts had greater kidney weight to body weight ratios in comparison to fetuses from CON gilts on d 60 of gestation (*P* < 0.05; Fig. 3B). There were no significant effects of treatment on the ratios of spleen, liver, and fetal biceps-femoris weight to body weight (*P* > 0.05); however, mean weigh of fetuses, as well as weight of kidney, liver, and biceps-femoris were greater for fetuses in the fall replicate than the summer replicate on d 60 of gestation (*P* < 0.0001; Fig. 4). Mean spleen and liver weight for fetuses was greater (*P* < 0.001) in the summer than fall replicate on d 90 of gestation shown in Fig. 4. There was no significant effect of treatment on litter size, fetal CRL or fetal body weight (*P* > 0.05).

### Effects of Cr supplementation on fetal muscle fiber development

**Fig. 5:**
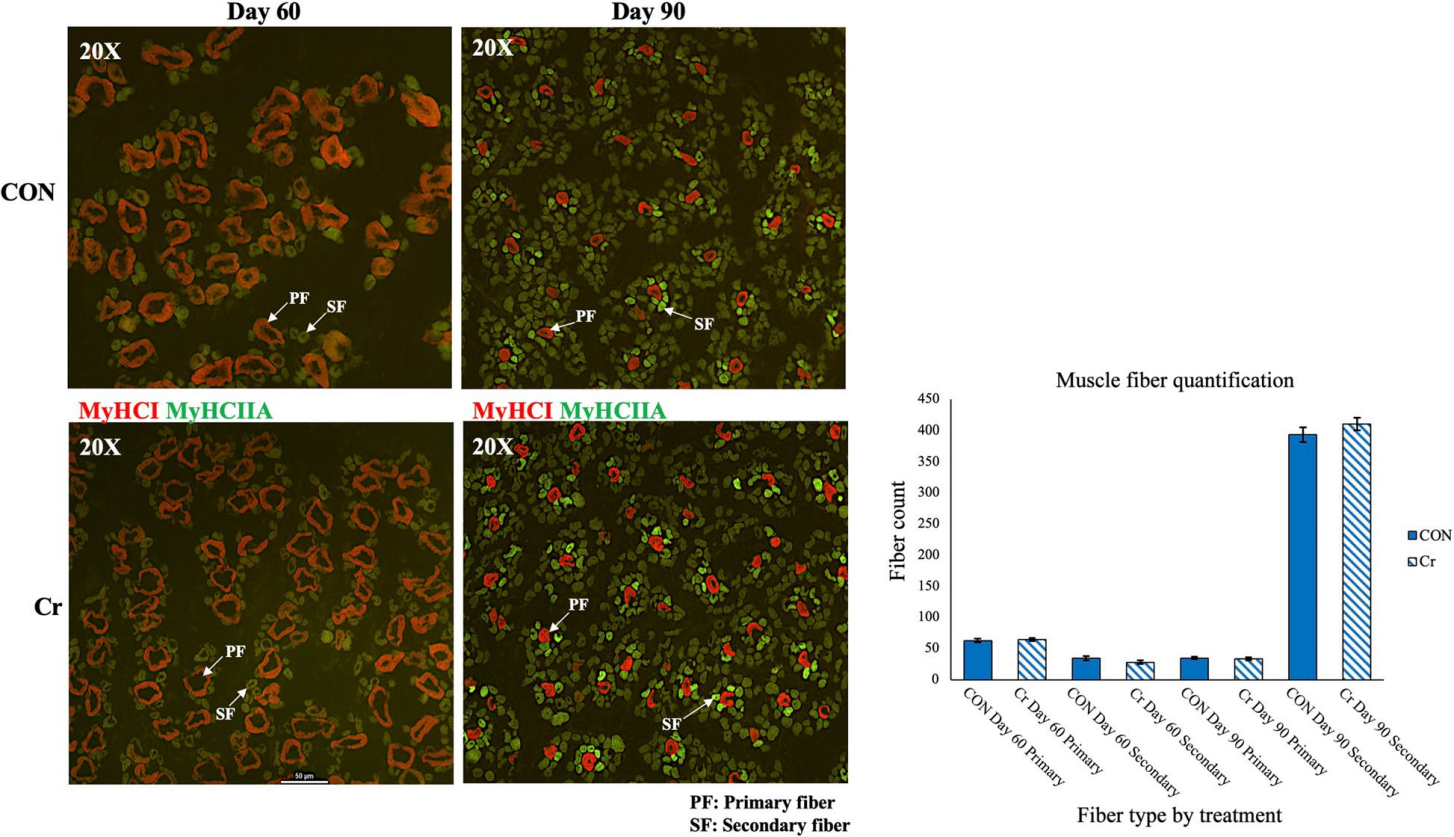
Immunofluorescence localization of MyHCI to detect primary skeletal muscle fibers and MyHCIIa to detect secondary skeletal muscle fibers. Biceps-femoris sections from replicate 1 were incubated overnight at 4 °C with primary antibodies at a 1:500 dilution for myosin heavy chain Type 1 to determine primary muscle fibers, and myosin heavy chain Type IIa to determine secondary muscle fibers. Five non-overlapping images were taken, then primary and secondary muscle fibers were counted blind to treatment by one individual. There was no significant (*P* > 0.05) treatment effect on primary or secondary muscle fiber counts for fetuses from either d 60 or d 90 of gestation. Values are mean $\pm$ SEM (*n* = 24 CON d 60 fetuses, *n* = 30 Cr d 60 fetuses, *n* = 24 CON d 90 fetuses, *n* = 28 Cr d 90 fetuses)

Primary and secondary fetal skeletal muscle fibers were identified and quantified in fetal biceps-femoris samples from replicate 1 using immunofluorescence for localization of MyHCI (primary skeletal muscle fibers) and MyHCIIa (secondary skeletal muscle fibers). Muscle fiber typing analyses of primary and secondary fetal muscle fibers are represented in Fig. 5. There was no effect of treatment (*P* > 0.05) on primary or secondary muscle fiber counts for fetuses from d 60 and d 90 of gestation. Muscle fiber typing results demonstrated that by d 60 of gestation primary skeletal muscle fibers were fully formed and by d 90 of gestation secondary skeletal muscle fibers are fully formed.

### Effects of Cr supplementation on expression of candidate proteins in endometria and fetal muscle

**Fig. 6:**
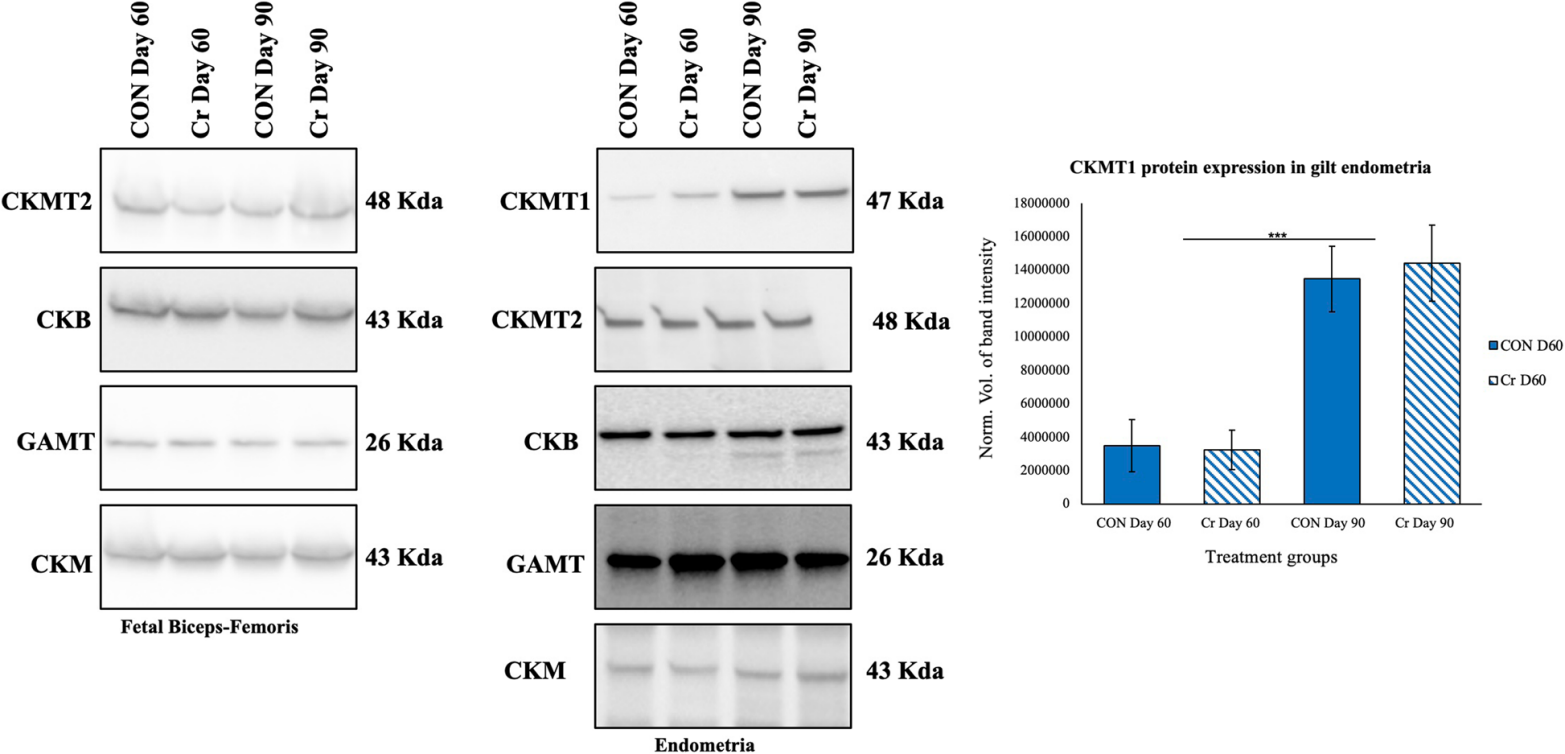
Representative images of candidate proteins expressed in endometria and fetal biceps-femoris muscle determined using Western blot analyses. Endometrial and fetal biceps-femoris samples from replicate 1 used for Western blot analyses. Primary antibodies used were rabbit polyclonal antibody CKMT1A (15346-1-AP, Protein Tech, Rosemont, IL, USA), CKMT2 (ab121450, Abcam, Cambridge, UK); CKMM (ab151465, Abcam); CKB (ab92452, Abcam); and GAMT (ab126736, Abcam), and all primary antibodies used at 1:500 dilution. Results of quantification of GAMT, CKB, CKM, CKMT1, and CKMT2 in endometria revealed no effects of treatment (*P* > 0.05). However, there was an effect of day of gestation with endometrial samples from d 90 of gestation having greater expression of CKMT1 than for samples from d 60 of gestation (*P* < 0.0001), values are mean ± SEM (*n* = 4 CON d 60 gilts, *n* = 5 Cr d 60 gilts, *n* = 4 gilts CON d 90 gilts, and *n* = 5 Cr d 90 gilts). There were no treatment effects on the expression of GAMT, CKB, CKMT2, and CKM proteins in fetal biceps-femoris muscle (*P* > 0.05). CKMT1 was not detectable in fetal biceps-femoris muscle (*n* = 1 CON d 60 fetuses, *n* = 1 Cr d 60 fetuses, *n* = 16 gilts CON d 90 fetuses, and *n* = 20 Cr d 90 fetuses)

Expression of key enzymes of the Cr-CK-PCr system was determined for endometrial and fetal biceps-femoris muscle using Western blotting to determine effects of dietary Cr supplementation in gilts. These candidate proteins were GAMT, CKB, CKM, CKMT1, and CKMT2. Results of quantification of GAMT, CKB, CKM, CKMT1, and CKMT2 in endometria revealed no effects of treatment (*P* > 0.05). However, there was an effect of day of gestation with endometrial samples from d 90 of gestation having greater expression of CKMT1 protein in comparison to endometrial samples from d 60 of gestation (*P* < 0.0001). There were no treatment effects on expression of GAMT, CKB, CKMT2, and CKM proteins in fetal biceps-femoris muscle (*P* > 0.05). CKMT1 was not detectable in fetal biceps-femoris muscle. These results are shown in Fig. 6. Representative total protein gels demonstrating consistent loading of gilt endometria and fetal biceps-femoris samples are shown in Fig. S1.

## Discussion

The important physiological roles of the Cr-CK-PCr system, specifically its ATP buffering capacity, have been clearly demonstrated in mammalian skeletal and cardiac muscles, brain, within the uterine-placental interface, conceptus, and spermatozoa, as these tissues/cells have high and fluctuating ATP demands [9, 21]. Within the body, Cr released by the liver and pancreas is transported to target tissues such as skeletal muscles, brain, and testes, and 95% of Cr is found in skeletal muscles [9]. The Cr-CK-PCr system has been investigated rigorously in muscle tissues due to its important roles in both skeletal and cardiac muscle functions [22, 23]. As the Cr-CK-PCr system is crucial for muscle functions, Cr supplementation during exercise promotes greater gains in strength, fat-free mass, and athletic performance during high intensity exercise tasks [24]. A study of dietary supplementation of 5 g Cr monohydrate given four to six times daily to adult humans resulted in an increase in total Cr content in the quadriceps femoris muscle, which emphasizes the roles for the Cr-CK-PCr system in muscle function [25]. Therefore, analyses of total Cr content in the fetal biceps-femoris muscle may reflect a similar result, although no treatment effect was revealed in the number or ratio of primary and secondary muscle fibers.

Besides skeletal muscles, the Cr-CK-PCr system is important for proper brain functions as the brain accounts for a substantial proportion (e.g., about 20% in adult humans) of whole-body energy expenditure [26]. Within the central nervous system (CNS), both AGAT and GAMT are expressed and the brain may be able to synthesize Cr [27]. The expression of CK in both adult and developing human brains suggests that the Cr-CK-PCr system has important roles in CNS functions [28]. Results of the present study indicated a significant effect of Cr supplementation to gilts to increase the ratio of brain weight to body weight on d 90 of gestation, in comparison to fetuses from CON gilts, emphasizing the concept that the Cr-CK-PCr system has critical roles in fetal brain development.

The presence of the Cr-CK-PCr system and its roles during gestation have been reported for the female reproductive tracts of humans, mice, sheep, pigs, and rodents [11, 21, 29–32]. The evidence of an active Cr-CK-PCr system in uterine tissues and changes in expression of its components during pregnancy underscores the importance of its ATP buffering capacity in supporting a successful pregnancy [21, 32]. This study revealed that expression of the CK isoform, CKMT1, changes during gestation in swine endometria with significantly greater expression on d 90 than d 60 of gestation. A previous study in sheep identified *CKMT1 *mRNA by PCR and localized CKMT1 protein to the endometrial LE and GE of pregnant sheep [11]. As the LE is instrumental in hemotrophic support of the developing fetus, and the GE is essential for histotrophic support of the fetus in both sheep and pigs [33, 34].

The expression of isoform CKMT1 has been shown to correlate with the oxidative capacity of muscle and its expression is greater in cardiac muscle than in fast-twitch skeletal muscle [9]. This may explain why CKMT1 was not detected in the fetal biceps-femoris muscle [9]. Expression of CKMT2 protein in endometria was not affected by day of gestation, but CKMT2 is known to be co-expressed with CKM in muscle and, in this study, there was stable expression of CKMT2 even though CKMT1 protein expression was absent in these samples of muscle [35]. During pregnancy, the uterine environment may impact fetal development and the occurrence of IUGR. This concept of the fetus’s environment impacting fetal development was demonstrated as mean weight of the fetal body, kidney, liver, and biceps-femoris were greater in the fall replicate than summer replicate on d 60 of gestation. In contrast, mean spleen and liver weight from fetuses was greater in the summer replicate than fall replicate on d 90 of gestation. These results support the concept that environmental factors such as heat stress and season impact fetal development [3, 4].

As the Cr-CK-PCr system has been identified in reproductive and developing fetal tissues, it may have important roles in fetal growth and development through impacting the uterine environment. Also, although there was no effect of treatment on the incidence of IUGR in this study, maternal under-nutrition nutrition leading to fetal under-nutrition results in impaired fetal growth [36]. Therefore, the concept of fetal programing through maternal nutrition and its impacts on the uterine environment may be a method of preventing IUGR in swine. The instance of IUGR in fetuses leads to fetal adaptations in skeletal muscle as evidenced by a decrease in the proportion of oxidative muscle fibers and impairment of myoblasts in skeletal muscle fibers [37]. As skeletal muscle is important for overall fetal development, understanding the roles of the Cr-CK-PCr system in developing skeletal muscle is vital and may lead to new methods of prevention of IUGR in swine. This study demonstrated expression of candidate proteins GAMT, CKM, CKB, and CKMT2 in the fetal bicep-femoris tissue, which emphasizes that the Cr-CK-PCr system is important for proper development of skeletal muscle. A previous study indicated that dietary supplementation of Cr in gilts during the last week of gestation reduced the incidence of piglets with low birth weight and improved myelination of the brain in piglets [38]. Therefore, Cr supplementation and components of the Cr-CK-PCr system may have important preventative and therapeutic roles in fetal growth and development.

## Conclusions

The hypothesis of this study was that dietary Cr supplementation for gestating gilts would enhance fetal development, reduce the occurrence of IUGR, and increase numbers of primary and secondary muscle fibers in fetal skeletal muscle. This study also evaluated the impacts of season as results from the two replicates demonstrated that environmental factors such as season and heat stress had impacts on fetal growth and organ development. This was demonstrated as mean weight of fetuses and weight of kidney, liver, and biceps-femoris were greater for the fall than summer replicate on d 60 of gestation. Further, spleen and liver weight from fetuses was greater in the summer replicate than fall replicate on d 90 of gestation. Western blot analyses indicated that major components of the Cr-CK-PCr system are expressed in both endometria and fetal biceps-femoris muscle, confirming that the Cr-CK-PCr system is important for fetal development. There was an effect of day of gestation on CKMT1 expression in endometria with values on d 90 of gestation being greater than those on d 60 of gestation. This result emphasizes the importance of the Cr-CK-PCr system in late gestation. This study also demonstrated that fetuses from Cr supplemented gilts had a greater brain weight to body weight ratio on d 90 of gestation, in comparison to fetuses from CON treated gilts, and that fetuses from Cr supplemented gilts had greater kidney weight to body weight ratios in comparison to fetuses from CON treated gilts on d 60 of gestation. These results provided further evidence that the Cr-CK-PCr system has important roles in the development of the fetal brain and kidney. Collectively, these results identified the presence of the Cr-CK-PCr system in endometria of gilts and fetal skeletal muscle during gestation and indicated that dietary supplementation of Cr for gestating gilts affects development of brain and kidney during fetal development. Further investigations into how dietary Cr supplementation for pregnant gilts affects concentrations of circulating Cr and PCr in fetuses, as well as the expression of genes related to the Cr-CK-PCr system may reveal the underlying mechanisms of how the Cr-CK-PCr system impacts fetal development.

## Supplementary Information

Additional file 1: Fig. S1. Representative images of loading of total protein onto gels demonstrating consistent loading during Western blot analyses of proteins from endometria of gilts and fetal biceps-femoris samples from fetuses.

## Data Availability

The datasets used and/or analyzed during the current study are presented and available from the corresponding author upon reasonable request.

## Abbreviations

ADP: Adenosine diphosphate
AGAT: Arginine-glycine amidinotransferase
CK: Creatine kinase
CKB: Brain type creatine kinase
CKM: Muscle type creatine kinase
CKMT1: Mitochondrial type 1 creatine kinase
CKMT2: Mitochondrial type 2 creatine kinase
CL: Corpora lutea
CNS: Central nervous system
Cr: Creatine
CRL: Crown-rump length
GA: Guanidinoacetate
GAMT: Guanidinoacetate-N-methyltransferase
IUGR: Intrauterine growth restriction
MyHC: Myosin heavy chains
OCT: Optimal cutting temperature
PCr: Phosphocreatine
SLC6A8: Solute carrier family 6 member 8
