# Supplementary figures and images for: Effects of dietary supplementation of creatine on fetal development in gilts at d 60 and d 90 of gestation
Source: J Anim Sci Biotechnol. 2025 Mar 1;16:31. doi: 10.1186/s40104-025-01166-0 (PMC11871691; doi:10.1186/s40104-025-01166-0)

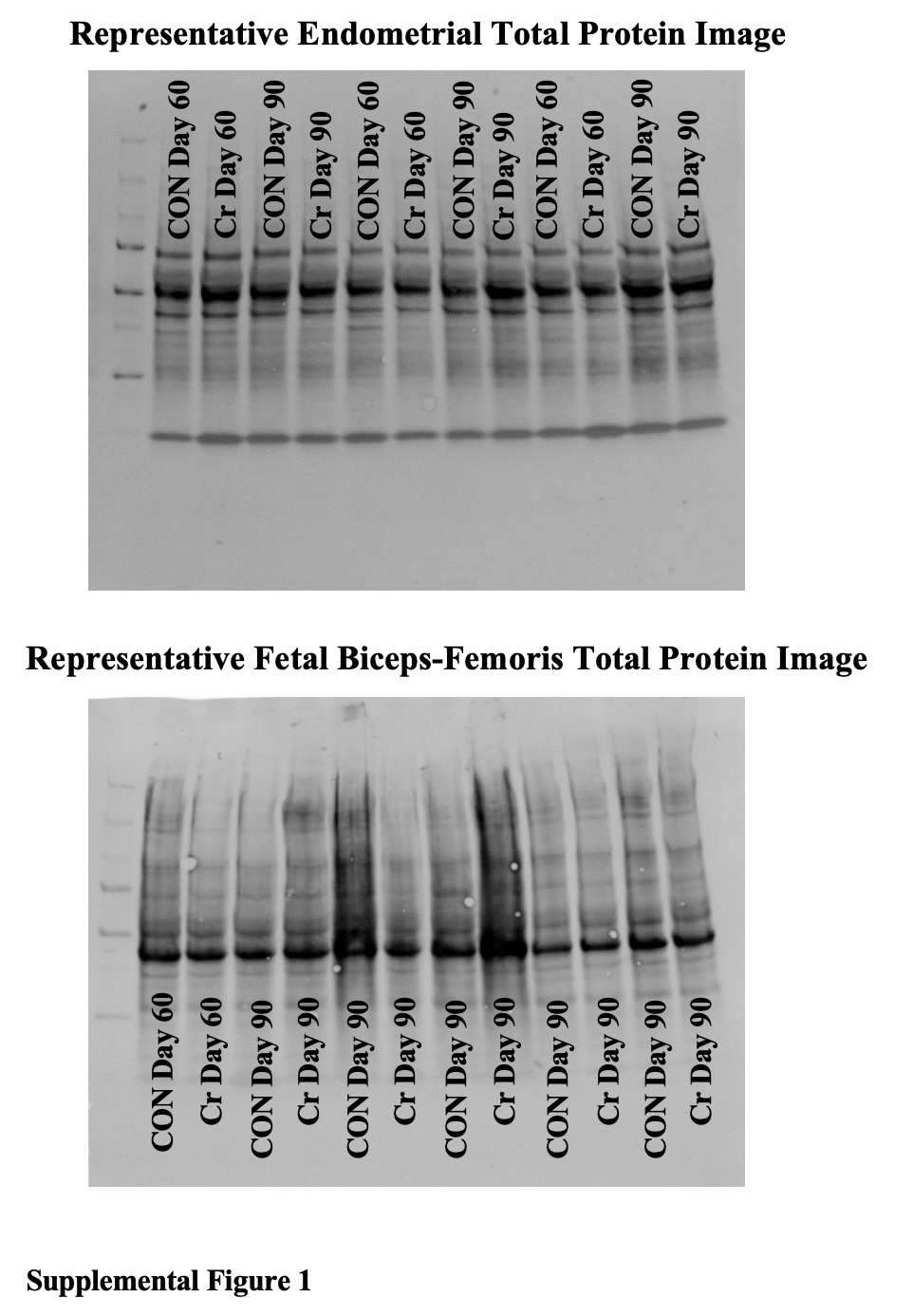

Supplement: Supplementary file 1 — Additional file 1: Fig. S1. Representative images of loading of total protein onto gels demonstrating consistent loading during Western blot analyses of proteins from endometria of gilts and fetal biceps-femoris samples from fetuses. [file 40104_2025_1166_MOESM1_ESM.jpg]
